# Speed Training Practices of Brazilian Olympic Sprint and Jump Coaches: Toward a Deeper Understanding of Their Choices and Insights (Part II)

**DOI:** 10.5114/jhk/174071

**Published:** 2023-10-27

**Authors:** Irineu Loturco, Tomás T. Freitas, Santiago Zabaloy, Lucas A. Pereira, Túlio B. M. A. Moura, Victor Fernandes, Valter P. Mercer, Pedro E. Alcaraz, Adam Zając, Chris Bishop

**Affiliations:** 1 NAR—Nucleus of High Performance in Sport, São Paulo, Brazil.; 2 Department of Human Movement Sciences, Federal University of São Paulo, São Paulo, Brazil.; 3 Department of Sport, Health, and Exercise Science, University of South Wales, Pontypridd, Wales, United Kingdom.; 4 UCAM Research Center for High Performance Sport, UCAM Universidad Católica de Murcia, Murcia, Spain.; 5 Facultad de Deporte, UCAM Universidad Católica de Murcia, Murcia, Spain.; 6 Faculty of Physical Activity and Sports, University of Flores, Buenos Aires, Argentina.; 7 Institute of Sport Sciences, The Jerzy Kukuczka Academy of Physical Education in Katowice, Katowice, Poland.; 8 London Sport Institute, Middlesex University, London, United Kingdom.

**Keywords:** athletic performance, sprint velocity, sprint speed, track and field, elite athletes

## Abstract

This is the second article in a three-article collection regarding the plyometric, speed, and resistance training practices of Brazilian Olympic sprint and jump coaches. Here, we list and describe six out of the ten speed training methods most commonly employed by these experts to enhance the sprinting capabilities of their athletes. Maximum speed sprinting, form running, resisted sprinting, overspeed running, uphill and downhill running, and sport-specific movement methods are critically examined with reference to their potential application in different sport contexts. In an era when sprint speed is of critical importance across numerous sports, practitioners can employ the methods outlined here to design efficient training programs for their athletes.

## Introduction

Speed is a key and decisive component in many sports (Jeffreys, 2013; Meyers et al., 2017). In general terms, running speed can be defined as the product of the stride rate and stride length (Nummela et al., 2007). Despite this apparent simplicity, sprinting has been invariably described as a complex skill that relies on a number of physical, technical, and mechanical aspects (Čoh et al., 2010; Loturco et al., 2019a). Recently, a considerable amount of research has been dedicated to investigating the main determinants of elite sprint performance (Gleadhill and Nagahara, 2021; Loturco et al., 2019a; Morin et al., 2012), which provides a solid foundation for the conception of novel and more effective speed training strategies (Loturco et al., 2019a; Janowski et al., 2017; Meyers et al., 2017). Nevertheless, the improvement of speed-related capacities remains a great challenge for coaches and sport scientists, who often question their own competence and capabilities to develop faster athletes (Haugen, 2017; Haugen et al., 2019; Loturco et al., 2023c). Although part of this awareness may stem from the lack of optimal conditions (e.g., congested training and match schedules, time constraints, etc.) for adopting and implementing the best and most effective training methods in elite sports, it is also important to acknowledge that a considerable gap still exists between science and practice in high-performance environments (Haugen et al., 2014, 2019).

Haugen et al. (2019) outline some of the possible explanations for these inconsistencies and discrepancies, being related to the assessment and examination of isolated sprint-derived variables (e.g., step length, step rate, flight/aerial time) under highly-controlled conditions, which greatly differ from the integrated and comprehensive speed training programs commonly utilized by coaches within different sport contexts. Added to this, the difficulties and concerns that naturally arise when applying certain specific training strategies (e.g., use of heavy loads or exercises with greater eccentric demands on days preceding official competitions or as a means to induce post-activation potentiation enhancement before or during sport-specific sessions) may contribute to keeping practitioners away from techniques and research questions that they consider unfeasible or of low-priority. Among several factors, including the limited trainability of speed-related qualities (Grazioli et al., 2023; Loturco et al., 2023a), this may explain why improvements in sprinting speed are minimal or even inexistent throughout the annual training season—a phenomenon that appears to affect athletes of different levels, sports, and age-categories (Gabbett, 2005; Haugen, 2017; Haugen et al., 2018; Loturco et al., 2023a; Zabaloy et al., 2022b).

Indeed, there is a clear need for more applied research and extensive analysis on this topic. Undoubtedly, a detailed examination of speed training methods employed by recognized experts in this field (e.g., professional track and field coaches) may be an important step toward the development of novel and more effective strategies for improving sprint performance in athletes of various sports. Following this rationale, the current article describes and critically examines the speed training practices employed by Brazilian Olympic sprint and jump coaches across the different phases of the competitive season, with emphasis on training programming, exercise selection, and potential training adaptations. The practices detailed here were part of an original survey study recently published in the *Journal of Human Kinetics* (Loturco et al., 2023b).

## Speed Training Programming

Overall, elite sprint and jump coaches utilize a comprehensive range of methods and strategies to optimize sprint speed (Table 1), while also focusing on improving the essential capabilities that form the foundation of sprinting and power performance. These critical attributes involve the simultaneous development of various physical qualities and skills, such as maximum strength and power capacities, speed endurance, “total body-power”, and speed technique (Tables 2 and 3) (Loturco et al., 2023b).

**Table 1:** Speed training methods, frequency of utilization among Olympic sprint and jump coaches, and the main objectives for using each method.

Training method	Frequency of utilization	Main objectives (Development of)
Maximum sprinting speed	84%	Sprint speed
Form running	74%	Sprint technique
Plyometrics	74%	Jump ability/Power/Injury prevention
Resisted sprinting	68%	Acceleration ability/Sprint speed
Overspeed running	58%	Sprint speed
Uphill/Downhill running	53%	Acceleration ability/Sprint speed
Resistance training	42%	Strength and power
Sport-specific movements	37%	Sprint technique
Complex training	21%	Strength and power
*Interval training	16%	Speed endurance

* **Note:** In the initial study, a small percentage of coaches (16%) reported using “interval training” as a method to enhance speed development. Given that the effects of this method are primarily associated with the cardiovascular system and the development of speed endurance, we chose not to include interval training in our discussion topics.

**Table 2:** A typical speed training program followed by an Olympic sprinter during the preparatory and competitive periods.^#^

	Preparatory period	Competitive period
**Exercises** **(Weekly frequency)**	*Maximum sprinting speed (1 x) Resisted sprinting (1–2 x) Uphill running (1–2 x) Sport-specific movements (1–2 x) Plyometrics (2–3 x) Form running (4–5 x) Resistance training (3–4 x)	**Maximum sprinting speed (2 x) Overspeed running (1–2 x) Plyometrics (1–2 x) Form running (3–4 x) Uphill/Downhill running (1–2 x) Resisted sprinting (1 x) Resistance training (2–3 x)

* sprints performed over shorter distances (i.e., 40–50 m)

** sprints performed over longer distances (i.e., ≥ 60 m)

# Note: Speed training programs elaborated by two Brazilian National Team Olympic coaches, with extensive international experience, including multiple participations in World Athletics Championships and Olympic Games.

**Table 3:** A typical speed training program followed by an Olympic long jumper during the preparatory and competitive periods. ^#^

	Preparatory period	Competitive period
**Exercises** **(Weekly frequency)**	Resisted sprinting (2–3 x) Uphill running (1–2 x) Sport-specific movements (2 x) Plyometrics (3 x) Form Running (5–6 x) Resistance training (3–4 x)	Maximum sprinting speed (1–2 x) Overspeed running (1–2 x) Plyometrics (2 x) Form running (5–6 x) Uphill/Downhill running (1 x) Resisted sprinting (1 x) Resistance training (2–3 x)

# Note: Speed training programs elaborated by two Brazilian National Team Olympic coaches, with extensive international experience, including multiple participations in World Athletics Championships and Olympic Games.

As expected, “maximum speed sprinting” is the method most commonly used by Olympic sprint and jump coaches, with a trend toward the utilization of longer distances (i.e., from 40 to 120 m) and higher training frequencies (i.e., from 1–2 to 2–3 times/week) from the preparatory to the competitive period. A similar trend is observed for overspeed drills (including downhill running), a pattern that, as detailed later, may be related to the inherent risks involved in maximal sprint training (e.g., hamstring injuries) (Engebretsen et al., 2010; Loturco et al., 2016), an aspect that can be even more problematic when these strategies are implemented at the initial phases of the season (i.e., pre-season) (Moreno-Pérez et al., 2022). In contrast, methods and strategies implemented with the main objectives of improving acceleration ability (e.g., resisted sprinting) and developing strength-power qualities (i.e., resistance training) present an opposite trend: a shift from higher to lower frequencies of use (i.e., from 2 to 1 times/week and from 3–4 to 2–3 times/week) and from higher to lower intensities (i.e., from 20–50% of body mass [BM] to 2.5–20% BM and from 90 to 40% of one-repetition maximum [1RM]) from the preparatory to the competitive period, for resisted sprinting and resistance training methods, respectively.

Plyometric exercises are regularly prescribed over the entire training season, after resistance training sessions (either on alternate days or immediately after) or as components of complex training sessions (Loturco et al., 2023b, 2023d). In addition, it is interesting to note a tendency for reduced training frequency (i.e., from 3 to 2 times/week) as the season progresses. Nonetheless, Brazilian Olympic sprint and jump coaches do not always follow a standard and uniform pattern for adjusting plyometric training content. As previously published, this content is usually tailored according to the needs and characteristics of athletes. Therefore, and as an example, if a given sprinter has a clear necessity to optimize top-speed qualities, they can perform vertically-oriented jumps for developing fast stretch-shortening cycle (SSC) ability (e.g., drop jumps with a focus on minimum contact time) from the earlier stages of the annual training season (Loturco et al., 2023b, 2023d; Pedley et al., 2017). On the other hand, sprinters with the primary intention of enhancing acceleration performance can continue to predominantly execute horizontally-oriented jumps (e.g., traditional bounding) throughout the season (Loturco et al., 2023a, 2023b, 2023d), which also exhibit greater specificity to acceleration characteristics by virtue of having longer contact times. A similar rationale is applied to the prescription of “form running” (i.e., technical drills) which is, to some extent, presumable, as these exercises are generally categorized as “sprint-specific drills” and implemented with the main purpose of “improving sprinting technique” (Healy et al., 2021; Loturco et al., 2023b). Hence, track and field coaches tend to select these exercises according to the similarity between the technical drill and the sprinting action (i.e., sprint movement or muscle activation pattern) being considered.

Sport-specific movements (e.g., sprinting actions or technical drills with added resistance) and interval training strategies appear as “complementary training activities” and both tend to follow a very individualized approach, but with a marked difference. Specifically, interval training is more commonly applied within the preparatory period, with the primary objective of developing speed endurance (Okudaira et al., 2019). However, sprinters who regularly experience meaningful detriments in performance due to fatigue in the final phases of sprint races or those specialized in longer distances (e.g., 200- and 400-m dash), may perform a higher and more frequent number of interval training sessions, “*even during the competitive period*” (response collected from open-ended questions). Conversely, sport-specific movements do not seem to follow a clear and consistent path of prescription, with sprint and jump coaches utilizing, for example, “*weighted forearm garments*” for “*optimizing arm action technique*” and “*weighted vests*” for “*improving vertical force application*” (responses collected from open-ended questions) in a wide variety of sprint-specific drills (e.g., maximal and submaximal sprint bouts), executed during different phases of the season. These exercises are often prescribed according to the subjective perception of coaches, with the main intention of enhancing specific aspects of sprinting technique (Loturco et al., 2023b, 2023d).

## Speed Training Methods

### Maximum Sprinting Speed

Sprint performance is a multifactorial phenomenon that relies on many different physical and physiological factors, such as the rate of force development, muscle power, maximum dynamic strength, and anaerobic power (Cronin and Hansen, 2005; Loturco et al., 2019a). Thus, it comes as no surprise that coaches who work closely with elite sprinters and jumpers consistently employ a diverse range of strategies to ensure the optimal development of sprinting speed (Loturco et al., 2023b). Among this variety of training approaches, maximum speed sprinting (MSS) continues to be the primary method used by these coaches to improve overall sprint performance (Table 3). This is somewhat evident, because in track and field disciplines such as sprinting and jumping events, the ability to sprint faster is directly related to competitive success (Loturco et al., 2023b, 2023d). However, it is worth noting that coaches from various team-sports (e.g., rugby union, rugby league, American football, and hockey) (Haro et al., 2020; Kugler and Janshen, 2010; Mann et al., 2022; Zabaloy et al., 2023), have also emphasized that maximal sprint drills play a key role in the preparation of their players. This further supports the notion that incorporating this training method is essential in almost any athletic training program, irrespective of the sport discipline.

In fact, a systematic review (Weldon et al., 2022) revealed that, in rugby, cricket, soccer, and basketball, ~80% of coaches regularly include MSS in their training routines. Moreover, Oliva-Lozano et al. (2023) recently highlighted the need to prepare elite soccer players for maximal intensity sprints, which, for these athletes, typically occur at distances shorter than 30 m and are performed at speeds of ~30 km/h. In practical terms, from a static starting position, football code athletes usually require 15 m to 40 m to achieve top speeds (Nicholson et al., 2022; Zabaloy et al., 2021). Nevertheless, from a rolling start (i.e., jogging or running at a moderate speed), these players may already be exposed to MSS patterns even when performing maximal sprints over short distances (e.g., ≤ 10 m) (Nicholson et al., 2022). For these reasons, athletes from a range of sports may benefit from MSS training, especially when these drills are performed across a broad range of distances and intensities, comprising the different phases of sprint running (e.g., acceleration and top-speed phases) (Cissik, 2005; Rumpf et al., 2016). Hence, if practitioners want to create speed training programs able to develop all the physical qualities required to repeatedly execute maximal and near-to-maximal sprints, they should comply with the principle of training specificity (Cissik, 2005; Kasper, 2019).

In line with the previous observations, coaches from rugby sevens, and Australian, American, and Gaelic football declared that the main rationale for sprint development in football code athletes is “to provide stimuli targeted at underpinning mechanical components of the neuromuscular system that determine sprint performance” (e.g., force-velocity-power-related output) as well as to optimize the technical efficiency of their athletes (i.e., orientation of forces applied onto the ground with increasing speed) (Nicholson et al., 2022). Nonetheless, it is essential to emphasize that, in most sports (especially in team sports), speed will come into play within a wide variety of game scenarios, in challenging and complex contexts (Caldbeck and Dos’Santos, 2022a; García-Sánchez et al., 2023; Jeffreys, 2017; Martínez-Hernández et al., 2023). In this sense, it is crucial to prepare players not only to cope with the expected and more controlled demands of linear sprinting, but also to effectively perform rapid and unexpected non-linear sprint tasks (e.g., directional changes involving acceleration and deceleration phases and curvilinear sprints) (Caldbeck and Dos’Santos, 2022a; Freitas et al., 2022b; Martínez-Hernández et al., 2023), skills that have been extensively researched and found to be strictly related to team-sport players’ performance (Dos’Santos et al., 2018; Freitas et al., 2022a, 2022b; Loturco et al., 2019b; Nimphius et al., 2018).

In addition, MSS training exposure should also be considered a critical aspect for both enhancing athletic performance and reducing the risk of suffering lower limb soft-tissue injuries (e.g., hamstring injuries) (Edouard et al., 2019; Malone et al., 2017; Nicholson et al., 2022). Importantly, the relative number and frequency of hamstring injuries in elite sports have been gradually increasing over the years (Ekstrand et al., 2016, 2022), which may be a direct result of increases in game speed and accumulated training loads (Aiello et al., 2023; Barnes et al., 2014; Dellal et al., 2015). Even so, curiously, this issue also affects elite track and field athletes who compete in different events (e.g., combined events, sprint races, marathon, and long-distance running) (Edouard et al., 2016). In fact, “muscle” and “hamstring muscle injuries” were the main types and categories of injuries diagnosed across sixteen international athletics championships, between 2007 and 2015, especially for athletes participating in “explosive power events” (i.e., faster athletes such as sprinters and jumpers). On the other hand, it has been shown that greater exposure to high percentages of MSS during training situations could offer a protective effect against hamstring injuries (when compared to lower exposure to MSS training situations) (Malone et al., 2017). Based on these findings and other biomechanical factors (e.g., unparalleled, and unique pattern of muscle activation), some authors have recommended the regular implementation of MSS training as a potential “vaccine” for reducing and mitigating the rate of subsequent muscle injuries, particularly, the incidence of hamstring muscle injuries (Edouard et al., 2019; Malone et al., 2017).

MSS training can also contribute to increasing the “speed reserve” (i.e., relative difference in terms of speed between submaximal and near-to-maximal sprints versus maximal sprints) (Loturco et al., 2020a; Vescovi, 2014), thus attenuating the negative impact of acute and chronic exposure to high-speed running distances across the competitive season (Aiello et al., 2023; Iatropoulos and Wheeler, 2023; Jokela et al., 2023). Indeed, previous studies have suggested and provided evidence that rapid and large increases in high-speed running volumes, compared to the volumes regularly performed by athletes, may significantly increase the likelihood of hamstring injuries (Duhig et al., 2016; Saw et al., 2018). From this perspective, athletes who can reach higher sprint speeds may experience lower-intensity efforts (in terms of maximal speed) across the season, particularly during official matches and technical-tactical training sessions, where it becomes challenging to control and predict high-intensity stimuli (Oliveira et al., 2021; Romero-Moraleda et al., 2021). In other words, faster athletes would also be more protected and better prepared to meet the ever-increasing, and sometimes excessive, demands of maximum speed present in modern sports.

In summary, Olympic sprint and jump coaches consistently rely on MSS training as a primary method to improve overall sprint performance. This method is widely used not only in track and field, but also in team sports, highlighting its relevance for practitioners with different perceptions, purposes, and backgrounds. Incorporation of MSS training into athletes’ programs, encompassing a range of distances and intensities, is essential for developing the physical and technical qualities required to perform maximal and near-to-maximal sprints effectively and repeatedly. Furthermore, MSS training plays a crucial role in reducing the risk of lower limb soft-tissue injuries. The implementation of MSS training can enhance the speed reserve and mitigate the negative effects of high-speed running exposure during the competitive season. Overall, MSS can be regarded as a simple, sport-specific, and highly effective method for preparing high-performance athletes. Coaches from various sports should consider incorporating MSS drills into their training programs, both to enhance performance and reduce the risk of muscle injuries in real-world sport settings.

### Resisted Sprinting

Sprint training methods may be divided into three different categories: *primary* (unresisted sprints), *secondary* (e.g., assisted sprints or resisted sprints), and *tertiary* methods (e.g., plyometrics, resistance training, and heavy and very heavy sleds) (Zabaloy et al., 2023). While unresisted sprints are commonly employed in various sports with different approaches and perspectives, other methods also have their own importance and serve specific objectives. Resisted sprinting, for example, is traditionally used to improve sprint speed and acceleration ability (Harrison and Bourke, 2009). Several implements are utilized during resisted sprints, which involve athletes running with an external overload (e.g., loaded sled, weighted vest, or parachute). Recently, the use of sled towing or weighted vests as training tools to enhance athletes’ acceleration and sprint speed has garnered significant attention from researchers and practitioners (Alcaraz et al., 2018; Zabaloy et al., 2023). Nonetheless, the effectiveness of the aforementioned training methods in developing these key physical qualities is still unclear and controversial (Alcaraz et al., 2018; Petrakos et al., 2016; Zabaloy et al., 2023). One important aspect to consider is that decreases in sprint velocity are dependent not only on the magnitude of the load, but also on the distance covered during resisted sprints (Pareja-Blanco et al., 2022a, 2022b; Zabaloy et al., 2022a). In practical terms, this means that higher loads, especially when combined with longer distances, can lead to greater decreases in sprint velocity and substantial disruptions in sprinting technique (Pareja-Blanco et al., 2022a, 2022b; Zabaloy et al., 2022a, 2023). The latter point highlights one of the main and most important considerations to be taken into account when designing resisted sprint training programs.

Resisted sprinting was commonly used by 68% of Brazilian Olympic sprint and jump coaches, which is somewhat comparable to the findings reported in rugby and football, where approximately 55% of coaches employ this strategy (Loturco et al., 2022; Zabaloy et al., 2022c). This method can replicate, from a broader perspective, the typical motor pattern observed in traditional sprints (Loturco et al., 2023b; Whelan et al., 2016), thereby promoting its regular utilization by practitioners as a speed-specific training strategy. Indeed, Whelan et al. (2016) reported that the majority of track and field coaches expressed the belief that speed drills should be tailored to (and thus reflect) the specific sprinting action. An addendum to the previously cited study (Loturco et al., 2023b) (Table 4) further revealed that Brazilian Olympic sprint and jump coaches predominantly employed two distinct loading prescription methods. Specifically, it was found that 47.4% of these coaches implemented a velocity decrement (% V_dec_) method, while 36.8% of them opted for a BM approach (% BM). In terms of sled loading conditions, it was observed that ~50% of Brazilian Olympic sprint and jump coaches utilized either light or moderate loads, which corresponded to a range of 2.5% to 10% V_dec_ or 2.5% to 20% BM and of 10% to 30%V_dec_ or 20% to 50% BM, respectively. None of these coaches implemented heavier loading conditions, such as those exceeding 30% V_dec_ or 50% BM. This also aligns with the findings of Whelan et al. (2016), as applying sled loads greater than 50% BM (~30% Vdec) heavily affects sprinting mechanics, posture, and technique (Pareja-Blanco et al., 2022a, 2022b; Zabaloy et al., 2022a, 2023), aspects that certainly influence the coaches’ choices.

**Table 4:** Coaches’ responses to sled loading prescription methods and magnitudes for resisted sprint training programming.

	Absolute (n)	Relative (%)
*Loading prescription methods*		
% Velocity decrement	9	47.4
% Body mass	7	36.8
Did not determine	2	10.5
Other	4	21.1
*Magnitude of sled loads*		
Light (2.5% to 10% V_dec_ or 2.5% to 20% BM)	9	47.4
Moderate (10% to 30% V_dec_ or 20% to 50% BM)	9	47.4
Heavy (>30 V_dec_ or >50% BM)	0	0
Other	1	5.2

V_dec_: velocity decrement; BM: body mass. “Other” responses included factors such as “perception of effort” and “video analysis of the movement” for sled loading prescription methods, and “it depends on the period of the season” for sled loading magnitude.

In order to avoid undesirable effects or maladaptation in either the short or long term, it is crucial to consider a number of factors when programming resisted sprint training sessions (Zabaloy et al., 2023). These factors include the distance and volume of the drills, implement being used (e.g., sled or weighted vests), alterations in sprinting mechanics (i.e., kinetics and kinematics), metabolic effects, and the period of the season (Loturco et al., 2023b; Zabaloy et al., 2023). By taking these factors into account during the programming process, practitioners can optimize the effectiveness of resisted sprint training, while minimizing the risk of negative outcomes. For example, when heavier sled loads are employed to enhance horizontal force production, there is a concomitant required increase in impulse (i.e., the product of force and time) (Bentley et al., 2016). This increase is accompanied by longer ground contact times and shorter flight times, which consequently lead to a gradual, and sometimes substantial, decrease in sprint speed (Pareja-Blanco et al., 2022a; Zabaloy et al., 2022a). In contrast, to achieve superior top speeds, proficient sprinters need to be capable of applying high levels of vertical force onto the ground under time-constrained situations (Clark and Weyand, 2014; Colyer et al., 2018), an ability that may be seriously compromised with significant reductions in sprint speed (and associated changes in sprint kinematics) (Zabaloy et al., 2023). This information may assist coaches in selecting the most appropriate loads to optimize sprint qualities, especially when considering that, in most sports, athletes rarely initiate sprints from a standing start (Filter et al., 2023; Zabaloy et al., 2023). In this regard, the use of lighter sled loads allows athletes to achieve higher sprint speeds within shorter timeframes and distances, which closely resemble the pattern observed in various sport-specific tasks.

Indeed, from a kinematic perspective, increasing loads during resisted sprinting affects the sprint technique in multiple ways and to varying degrees. As previously discussed, when the load is increased during sled towing, ground contact time and trunk inclination also increase, whereas stride length and flight time gradually decrease (Pareja-Blanco et al., 2022a; Zabaloy et al., 2022a). Furthermore, significant changes in joint angular kinematics at the hip, knee, and ankle joints, along with critical alterations in muscle activity patterns were observed with increasing loads in rugby players, with a trend toward more pronounced disruptions (i.e., differences compared to unloaded sprinting technique) under very-heavy loading conditions (i.e., > 30% V_dec_ or > 50% BM) (Pareja-Blanco et al., 2022a; Zabaloy et al., 2022a). Progressive increases in contact time and decreases in flight time throughout the gait cycle may also explain the decrements in sprint speed commonly observed during resisted sprinting with weighted vests (i.e., “vertically-based resisted sprints”) (Carlos-Vivas et al., 2019b; Cross et al., 2014), as well as when using other alternative methods of applying resistance (e.g., uphill running, elastic bands, parachutes, and sand surfaces, etc.). These changes in sprinting motion and technique will also depend on the type and magnitude of the applied resistance, which will be proportional to factors such as the inclination of the hill (with steeper hills causing greater disruptions) or the density of the sand. However, it is worth noting that these alternative strategies (i.e., uphill running and sand training) (Ebben et al., 2008; Okudaira et al., 2021; Pereira et al., 2023a, 2023b) have also demonstrated effectiveness in improving sprint performance and, thus, should be approached with the same rationale when considering the prescription of resisted sprint training. Unless secondary adaptations in the sprint technique or potential improvements in acceleration rates from static positions are being sought, light and light-to-moderate decrements should be prioritized over greater decrements in sprint speed (Zabaloy et al., 2023). Practitioners should rely on these principles to prescribe alternative forms of resisted sprints.

As such, regardless of the apparatus or surface used to create resistance, when programming resisted sprint training sessions in high performance sport settings, coaches should carefully consider the potential (positive and negative) effects of the loading condition. This aspect is of utmost importance, particularly because the majority of elite athletes adhere to a highly congested fixture schedule, leaving them with limited time to recover from one part of training (e.g., a sprint or a strength-power training session) before moving on to the next workout (e.g., a technical-tactical training session) (Haugen, 2017; Loturco et al., 2022; Nuñez et al., 2022). For example, it has been shown that heavy sled loads (i.e., 80% BM) resulted in greater performance impairment (i.e., increases in sprint time) and metabolic responses (i.e., higher blood lactate concentrations) compared to lighter sled loads (i.e., 20% BM) after exercise (Bachero-Mena et al., 2020). Although all loading conditions (i.e., ranging from 20 to 80% BM) allowed for complete recovery of sprinting ability after 24 hours of rest, this immediate decrease in sprint speed may compromise the subsequent demands and activities (Bachero-Mena et al., 2020). Moreover, it remains unclear if this “extra overload” offers any advantages over the lighter loading conditions or if these acute responses might negatively impact physical and technical performance in the long-term (Zabaloy et al., 2023). Likewise, Monahan et al. (2022) demonstrated that a “heavy sled training session” (V_dec_ = 22.7%) led to a higher heart rate, blood lactate concentration, and the rating of perceived exertion than a “light sled training session” (V_dec_ = 7.5%) at post-exercise. According to those authors, before designing resisted sprint training programs, coaches should be aware of the additional demands associated with heavy sled loads and how this might impact other training sessions, competitive performance, and total training loads. As explicitly stated in that later study “unless a clear advantage of heavy resisted sprint training over light resisted sprint training emerges in future studies, practitioners should consider whether heavy resisted sprint training is justified in their athletes and respective sports”. The experience and perception of Brazilian Olympic sprint and jump coaches concerning these aspects, combined with their extensive experience working with highly-specialized sprinters, could potentially elucidate their strong inclination towards using light and light-to-moderate sled loads. Despite the recent debates in the literature, these important insights can serve as valuable guidance for coaches interested in designing and implementing efficient resisted sprint training programs.

### Overspeed Running

Fifty-eight percent of Brazilian Olympic sprint and jump coaches regularly prescribe overspeed running (or assisted sprinting) for speed development (Loturco et al., 2023b). The technique involves the utilization of various types of equipment, such as elastic cords (Bartolini et al., 2011; Corn and Knudson, 2003; Hicks et al., 2023; Nealer et al., 2017), pulley-based towing systems (Kratky and Müller, 2013; van den Tillaar and Gamble, 2019), or motorized devices (Rakovic et al., 2018) to “pull” athletes forward while sprinting, with the aim of reaching supramaximal speeds (i.e., greater than those achieved by the athlete voluntarily and unassisted) (Hicks, 2017; Tufano and Amonette, 2018). When compared to unloaded or resisted sprinting, this methodology is considered to be more “velocity-oriented” given that it targets primarily at the velocity end of the force-velocity spectrum (Tufano and Amonette, 2018). Therefore, assisted sprinting can serve as a supplementary strategy to more conventional “force-oriented” training methods (e.g., resistance training or sled towing) in different sports (Hicks, 2017; Tufano and Amonette, 2018).

During overspeed running, there are important acute kinematic changes in the sprinting gait cycle. Specifically, the horizontal velocity of the center of mass, step length, and flight times have been shown to be significantly higher, and ground contact times significantly shorter, during assisted than during traditional sprinting (Corn and Knudson, 2003; Hicks, 2017; van den Tillaar and Gamble, 2019). These modifications contribute to achieving higher sprint speeds; however, caution is required when prescribing overspeed training. The improper determination of supramaximal stimulus (i.e., too much assistance) may result in: 1) athletes substantially altering their running mechanics and overstriding as a braking and protective strategy (Hicks, 2017); and 2) increased distances from the body’s center of mass to the foot’s center of mass (Clark et al., 2009; Corn and Knudson, 2003; Mero and Komi, 1987) which ultimately generate greater braking forces at ground contact (Clark et al., 2009; Mero and Komi, 1987). When combined, these factors may have an adverse effect on athletic performance, particularly in relation to sprinting technique. For these reasons, determining the optimal assisting force becomes crucial for practitioners aiming to prescribe effective overspeed training sessions.

In practical settings, prescribing overspeed training based on percentage increases in velocity is a common practice. Different authors (Cissik, 2005; Hicks, 2017) have already suggested that an adequate assisting force should prevent athletes from achieving speeds greater than 110% of their top-speed to avoid substantial disruptions in sprinting technique, which could be harmful to athletes (i.e., risk of falling) and hinder the proper development of sprint performance. With this in mind, Bartolini et al. (2011) analyzed the acute effects of using different levels of elastic cord assistance (i.e., equivalent to 10%, 20%, 30%, or 40% of body weight, in N) to determine the “optimal” assisted sprinting training conditions in collegiate female soccer players. Those authors utilized a crane scale to determine and adjust the elastic cord tension to achieve the intended assistance level (i.e., 10%, 20%, 30%, or 40% of body weight) and concluded that faster performances were observed up to 30% body-weight assistance; hence, this specific loading condition was considered as “optimal” and recommended as the upper limit for implementing effective overspeed training sessions (i.e., no improvement was noticed at 40% body-weight assistance when compared to 30%). The results from that investigation reinforce the notion that excessive assistance may not necessarily lead to higher sprint speeds.

Repeated exposure to overspeed stimuli has been shown to lead to significant improvements in sprint speed, similar to those obtained with resisted sprinting, but usually superior to unassisted sprinting alone (Lahti et al., 2020; Makaruk et al., 2019; Murray et al., 2017; Upton, 2011). For example, in a sample of female soccer players, a 4-week intervention involving assisted or resisted sprinting led to improved sprint speed and a greater acceleration rate (Upton, 2011). Conversely, the unloaded training group did not exhibit any meaningful change. This is line with the study by Hicks et al. (2023) which showed that the combination of unloaded and assisted sprinting (pulling force of 97.5 ± 15 N, corresponding to ~17% of body weight) resulted in meaningful gains in sprint performance (pre-post % changes from ~2.8 to 4.5%, for sprint distances between 5 and 20 m), whereas unloaded sprint training did not present any significant change in sprint speed in young team-sport athletes.

Apart from these interesting results, there are other promising aspects worth noting regarding assisted sprinting: 1) the maintenance of post-training adaptations (i.e., following a 3-week detraining period) has been reported to be greater after overspeed training compared to unloaded sprint training (Makaruk et al., 2019); and 2) there are no documented reports of increased muscle damage following this training method (Tufano and Amonette, 2018), as opposed to more “force-oriented” stimuli (i.e., resisted sprints) (Bachero-Mena et al., 2020), assuming that the level of assistance does not generate excessive braking forces at ground contact, given that higher eccentric overloading could result in greater amounts of muscle damage (Hody et al., 2019). This may have important implications for training programming, as it indicates that assisted sprint training could be recommended during the competitive phase of the season or before periods of reduced training volume (i.e., tapering) in order to preserve sprint capacity.

In conclusion, when properly prescribed, overspeed training can be safely used to complement more conventional methods of speed training, such as resisted sprinting or MSS training. From a practical perspective, it is recommended that coaches determine assistance loads based on percentage increases in sprint speed, keeping in mind that athletes should not exceed the maximal speed achieved within the target distance by more than 10%. As mentioned earlier, running speeds higher than this speed zone will acutely alter sprinting kinematics, to such an extent that it may be detrimental for performance or harmful to athletes. In terms of programming, overspeed training sessions may be prescribed during the in-season periods or even before phases of reduced training volume (i.e., tapering phase), as competitions approach.

### Uphill/Downhill Running

Uphill and downhill running are cost-effective and easy-to-implement speed training methods employed by a considerable percentage (53%) of Brazilian Olympic jump and sprint coaches (Loturco et al., 2023b). These methods involve utilizing natural or artificial gradients, or hills, to modify the load imposed on the athlete while sprinting (Ebben et al., 2008; Paradisis et al., 2009; Paradisis and Cooke, 2001). Conceptually, uphill and downhill running can be considered specific types of resisted and assisted sprinting, respectively (Hicks, 2017). However, as both methods are performed without any additional equipment (which enables their use with a large group of athletes simultaneously), it is not uncommon for practitioners to place them into their own “separate category” of exercises (Loturco et al., 2023b).

A critical aspect to consider regarding uphill or downhill running is that the amount of resistance/assistance and the acute sprinting stimulus will directly depend on the slope angle. Delaney et al. (2022) reported that, in uphill running, for every 1% increase in the inclination angle, sprinting speed decreases by ~2%, on average. Conversely, in downhill conditions, Ebben et al. (2008) demonstrated that running speed gradually increased with the slope gradient (i.e., 2.1º, 3.3º, 4.7º, 5.8º) up to a declination of 6.9º (i.e., the point of “diminishing return”). Thus, practitioners should be aware that not all slopes have the potential to induce positive adaptations in speed performance and that steeper surfaces will lead to greater alterations in sprinting mechanics (compared to horizontal sprinting) (Delaney et al., 2022; Ebben et al., 2008; Okudaira et al., 2021; Paradisis and Cooke, 2001).

Consistent with other overspeed training methods, downhill running has been shown to induce acute sprint kinematic adjustments (e.g., increased maximal running speed, stride length, and touchdown distance) and postural changes (e.g., an increased shank, knee and hip angle, a decreased thigh to thigh angle at touchdown, and a decreased shank and knee angle at the take-off) in comparison to flat surface sprinting, resulting in higher speeds (Ebben et al., 2008; Paradisis and Cooke, 2001), provided that the slope is not excessively steep and does not result in increased braking forces during ground contact (Tufano and Amonette, 2018). In addition, downhill running may lead to positive long-term speed-related adaptations in flat surface sprinting (e.g., higher step rates and shorter step times, resulting in faster running speeds) (Paradisis and Cooke, 2006). Therefore, downhill running can be considered a viable strategy for speed development.

When considering uphill running, conflicting results can be found in the literature regarding its effectiveness. On the one hand, there are reports of positive adaptations in some independent measures of athletic performance, such as strength (i.e., lower-body and back isometric strength), COD (i.e., 5-10-5 test completion time), and endurance performance (i.e., Yo-Yo Intermittent Recovery Test and 3-km time trial results), in semi-professional soccer players after a 6-week uphill sprint training protocol completed at a 7% gradient (Kavaliauskas et al., 2017). In contrast, Paradisis and Cooke (2006) observed no performance improvements following an uphill running intervention conducted on a specifically designed platform with a 3º slope. As such, and despite the acute kinematic changes detected during uphill running (e.g., altered lower body joint angles) that resemble certain essential aspects of the acceleration sprint phase, to date, it is not well-established whether this methodology is as effective as other resisted sprinting methods, such as sled towing. Thus, when deciding which strategy to utilize with their athletes, coaches should consider that: 1) sled towing might allow greater training individualization (i.e., loads are easier to adjust for each athlete), but requires more equipment and material resources; and 2) uphill running might be better suited for large group settings (i.e., multiple athletes may train at the same time), but manipulating the load is harder as it usually depends on the naturally available slopes (e.g., hills).

Uphill sprints can also be used in combination with downhill sprint training to optimize positive changes in speed performance. Indeed, different studies have demonstrated the superiority of uphill-downhill training interventions over traditional sprinting, or uphill and downhill sprinting alone (Bissas et al., 2022; Cetin et al., 2018; Paradisis et al., 2009; Paradisis and Cooke, 2006). These results may be explained by the fact that uphill-downhill sprinting combines resistive (i.e., when running uphill with a 3º slope sprint speed may decrease by ~3%) and supramaximal speed efforts (i.e., during downhill running on a track with the same 3º slope, sprint speed increases by ~8.5%) which may overload the neuromuscular system to a greater extent that when each of these methods is applied in isolation (Paradisis et al., 2009; Paradisis and Cooke, 2006). Nevertheless, the application of uphill-downhill training is greatly restricted in real-world contexts due to the scarcity of training facilities (or even natural structures) that can enable this type of training.

To conclude, both uphill and downhill running are alternative methods for speed development that can be implemented to artificially introduce additional resistance or increased sprint speed into speed-specific training programs. Regarding uphill running, practitioners should keep in mind that inclinations up to 10% lead to acute postural adjustments, resembling those observed in the late acceleration phase of sprinting, whereas steeper gradients replicate running mechanics more closely related to those seen in the early acceleration phase. When it comes to downhill running, slopes steeper than ~7º can lead to increased braking forces upon foot strike, which may have a negative impact on sprint performance. Hence, coaches should consider using slopes no steeper than 6º to enable athletes to adequately achieve their “supramaximal speeds”.

### Sport-Specific Movements

Sport-specific movements performed with an added resistance (e.g., weighted vests) are among the myriad of methods employed by practitioners from various sports for speed development (Macadam et al., 2022). Specifically, approximately 4 out of 10 Brazilian Olympic sprint and jump coaches (37%) reported using this training strategy with their athletes (Loturco et al., 2023b), with the majority indicating the use of “*weighted vests*” (response collected from open-ended questions). Despite some mechanical similarities (i.e., sprints performed with additional resistance), it is important to highlight that weighted vest sprinting and sled towing are distinct training methods that impose different types of overloading on the body (i.e., vertical versus horizontal overloading, respectively), thus eliciting different acute changes in sprint kinematics (Cronin et al., 2008). As such, the rationale for prescribing weighted vest sprinting and its perceived advantages with respect to sled towing rely on the possibility of: 1) loading linear sprints with less noticeable disruptions in movement mechanics (for the same relative load) as the load is directly placed “on the body” (Cronin et al., 2008); and 2) overloading sport-specific tasks typically performed during training and competition, such as COD drills and jumps (Carlos-Vivas et al., 2019a, 2020).

A recent investigation (Carlos-Vivas et al., 2019b) reported a range of mechanical and technical changes associated with weighted vest sprinting (i.e., decreased peak velocity, horizontal force production, and ratio of forces) when compared with unloaded sprinting in semi-professional soccer players. Importantly, the study revealed that these changes were considerably more pronounced with loads greater than 20% of BM. This notion is supported by another study (Cross et al., 2014) that noted that weighted vest sprinting, with either lighter or heavier loads (~11% and 22% BM, respectively), resulted in significantly lower flight times and step length during the top-speed phase of a treadmill sprint (in comparison to unloaded sprints performed on the same apparatus). Again, the study revealed that certain key kinetic variables (e.g., vertical ground reaction force) were only affected under the heavier loading condition. Therefore, it can be inferred that a lighter overload (e.g., ≤ 10% BM) should be used if the aim is to replicate the traditional sprinting technique without producing substantial changes in kinetic and kinematic variables.

Regarding long-term adaptations, only few studies (Carlos-Vivas et al., 2020; Clark et al., 2010; Rey et al., 2017; Rodríguez-Osorio et al., 2019) have examined the effects of overloading sport-specific movements using weighted vests. Overall, the results suggest that the use of this type of equipment does not lead to greater gains in linear sprint speed when compared to unloaded sprinting (Carlos-Vivas et al., 2020; Clark et al., 2010; Rey et al., 2017). It seems that, despite the acute changes in spatiotemporal variables induced by the use of weighted vests (Carlos-Vivas et al., 2019a, 2019b; Cronin et al., 2008), the repeated application of this stimulus over time may not translate into superior adaptations to sprint performance. Furthermore, the existing studies used a specific range of loads (i.e., from 10% to 50% BM), which limits the current understanding of the effects of using different loading conditions (e.g., ≤ 5% BM).

For COD ability, the use of weighted vests seems more promising. Previous studies (Carlos-Vivas et al., 2020; Rodríguez-Osorio et al., 2019) compared the effects of completing multidirectional sprints either unloaded or carrying a weighted vest, with loads ranging from 10% to 20% of BM, and found significantly greater improvements in the COD performance of soccer players under the latter condition. A plausible explanation may be related to the fact that, when using a weighted vest, athletes have to overcome higher eccentric loads during the braking phase of the directional changes (due to the greater momentum generated by the additional load) (Carlos-Vivas et al., 2020), which does not occur in unloaded COD training. Thus, considering these mechanical aspects and the well-documented effects of eccentric-based exercises on multidirectional performance (Chaabene et al., 2018; De Hoyo et al., 2016; Nygaard Falch et al., 2019), incorporating weighted vest sprint training can be seen as a viable and efficient strategy to enhance COD ability among athletes in various sports.

In summary, the implementation of sport-specific movements that mimic sprint or other sport actions (e.g., COD drills) is a practical and cost-effective training solution that, according to the literature, seems to have no additional ergogenic effect on linear sprint performance when compared to unloaded sprinting. Therefore, other sprint training methodologies (e.g., MSS and sled towing) should be preferred over weighted vest sprinting when the aim is to maximize linear sprint performance. Conversely, this method may be a suitable alternative to improve the ability to effectively execute directional changes, due to added load generating a greater eccentric stimulus, specifically in the vertical direction. To achieve these benefits, coaches are advised to prioritize light loading conditions (≤ 20% BM), as these loads cause minimal disruptions in movement technique and potentially provide a more effective transfer to athletic performance (Rodríguez-Osorio et al., 2019).

### Plyometrics

Brazilian Olympic sprint and jump coaches regard plyometric training as the third most important method for improving a range of sprinting skills (Loturco et al., 2023b). Approximately 90% of these coaches have stated that “speed development” is the primary reason for prescribing plyometric exercises throughout the season (Loturco et al., 2023b). Given this crucial importance and confirmed effectiveness, along with the wide variety of plyometric exercises cited and utilized by these practitioners, we decided to conduct a specific study on this topic (Loturco et al., 2023d). From the results of this study, it was found that: 1) hurdle jumps, drop jumps, box jumps, assisted jumps, standing long jumps, bounding, and multiple hops, are the most popular exercises among these coaches. These exercises are prescribed for different purposes (i.e., developing acceleration, top-speed, and jumping qualities) during both preparatory and competitive periods; and 2) overall, plyometric training sessions are more frequently prescribed during the preparatory period than during the competitive period (2–3 vs. 1–2 times/week, respectively). For a more comprehensive understanding of this specific topic, readers are encouraged to refer to the first article of this collection (Loturco et al., 2023d), already published in the *Journal of Human Kinetics*.

### Form Running

Form running (i.e., technical sprinting drills) is the second most commonly used method for speed development among Brazilian Olympic sprint and jump coaches (Bedini, 2012; Loturco et al., 2023b) (Table 3). Technical drills are widely prescribed in a variety of track and field disciplines because they closely resemble the movements executed during the various phases of sprint running (Whelan et al., 2016). Indeed, previous studies (Healy et al., 2021; Whelan et al., 2016) have already revealed that “similarity to sprinting” is perhaps the most important factor for sprint coaches when choosing and prescribing training exercises. It is worth noting, however, that these practitioners usually divide sprint races into different phases, each with distinct physical and technical demands (Collier, 2002; Jones et al., 2009; Thompson et al., 2009; Whelan et al., 2016). Hence, the accurate selection and prescription of these drills will depend on the specific movement pattern to be enhanced or skill acquisition to be achieved (e.g., body posture, trunk position and arm action during acceleration and top-speed phases).

The division of sprint phases lacks consensus among coaches and researchers, ranging from simpler, more general divisions into three main phases (i.e., “acceleration, transition, and full speed”) (Collier, 2002) to more complex and detailed classifications including six distinct phases (i.e., start, pure acceleration, transition, maximal velocity, speed maintenance, and finish) (Seagrave, 1996). Despite these controversies and different viewpoints, Jones et al. (2009) identified certain technical aspects and high-order constructs that are generally recognized and well-accepted by expert sprint coaches. To facilitate the training process and increase skill-learning retention, these practitioners break down sprint race into three broad phases: the start, the drive/pick-up, and the maintenance phase. The start phase encompasses constructs such as arm action, body position in the blocks, and the first step out of the blocks. Consequently, apart from track and field, technical drills related to this phase (e.g., block starts) have limited applicability in other sports.

In contrast, the drive/pick-up (acceleration) phase is unquestionably the most important phase to consider in the majority of sports (e.g., team-sports), where successful performances in numerous key situations (i.e., assists, goals, or scoring attempts) rely on the ability to achieve higher speeds over short distances (i.e., 10–20 m). Jones et al. (2009) also listed four critical elements highlighted by sprint coaches for this phase: arm action and leg extension (as primary constructs), and foot contact and posture (as secondary constructs). It is important to emphasize that there is a divergence between coaches and biomechanists concerning the actual contribution of arms to maximal sprints (Macadam et al., 2018; Thompson et al., 2009; Waters et al., 2019). However, in the coaches’ view, arms play a crucial role in improving balance and synchronization during sprinting. While no consensus exists on the optimal arm action to be adopted or developed, coaches generally agree that arm movements should vary between phases, with elbow flexion ~90° and shoulder flexion/extension occurring rapidly over shorter or relatively larger ranges of motion. Leg extension was mentioned as the second high-order construct, mainly because, during the acceleration phase, the full extension of hips and knees allows athletes to increase the total time of force application prior to the take-off, which, at least in theory, maximizes the force exerted onto the ground—a concept which is critical when overcoming inertia and building momentum.

**Figure 1:**
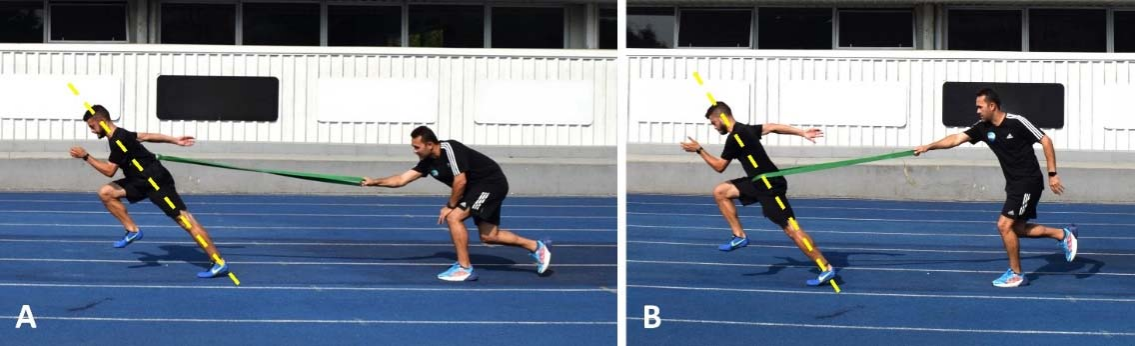
*. Acceleration drills with elastic bands: utilizing a vest to connect the band to the athlete’s trunk, thus allowing for greater trunk lean at the foot strike in order to resemble the sprinting posture adopted during the early acceleration phase (Panel A); with the elastic band directly attached to the athlete’s waist, thus positioning the trunk with a lower degree of inclination, resembling more closely the sprinting posture adopted during the mid-acceleration phase (Panel B). Both drills are intended to maximize horizontal force production, hip extension power, and improve sprinting posture during the acceleration phase. Sprint and jump coaches usually prescribe these drills over very short distances (e.g., 6–8 m).

**Figure 2:**
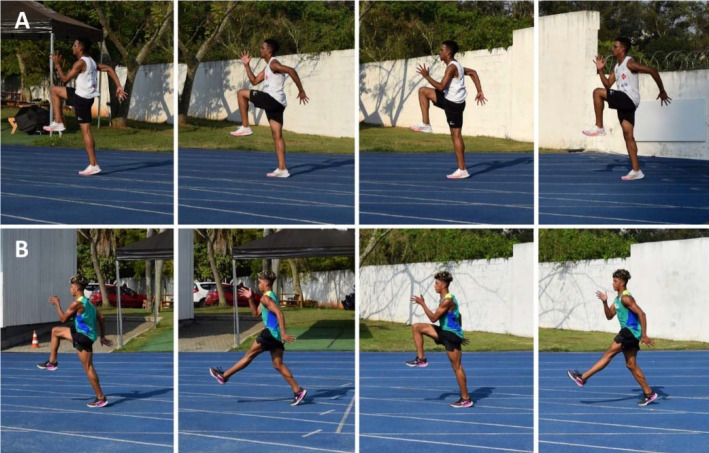
*. High knee skipping (Panel A) and unilateral high knee skipping with extension of the lower leg (Panel B). These forms of skipping can be utilized to improve foot contact technique, leg extension mechanics, and the hip position while maintaining a more upright position, closely resembling the sprinting posture adopted during the top-speed phase. Both technical drills involve a vigorous arm action, with the hands moving from the hip to shoulder height. Sprint and jump coaches usually prescribe these drills over very short distances (e.g., 6–8 m).

Despite being considered a secondary construct, foot contact or simply ground contact, which refers to the time from when the foot contacts the ground until it is lifted off again, is a widely researched and documented topic (Bushnell and Hunter, 2007; Coh and Tomazin, 2006; Cunningham et al., 2013; Thompson et al., 2009). It is well-established that ground contact time decreases and flight time increases with increasing speed, as a consequence of greater and progressive dependency on vertical force production (and smaller dependency on horizontal force production at higher speeds) (Coh and Tomazin, 2006; Colyer et al., 2018; Loturco et al., 2015; Moir et al., 2018; Wild et al., 2011). Therefore, this construct has significant implications for distinct sprinting phases (i.e., acceleration and top-speed), and should be addressed from different perspectives. When the primary goal is to maximize ground contact time (and horizontal force production), acceleration drills with elastic bands (Figure 1, Panels A and B) can be employed. Due to their technical and mechanical aspects, the same exercises may also be used to enhance hip extension power, making them effective for various purposes. Conversely, when the main objective is to improve foot contact technique (i.e., active foot landing) and leg extension, technical drills such as high knee skipping and high knee skipping with extension of the lower leg (Figure 2, Panels A and B) can be prescribed. Also in this case, the same exercises could be used to improve arm action, as they require vigorous movements (i.e., punching action from the hip to the shoulder height) of the upper limbs to balance the rapid swing of the legs (Mcfarlane, 1984).

**Figure 3:**
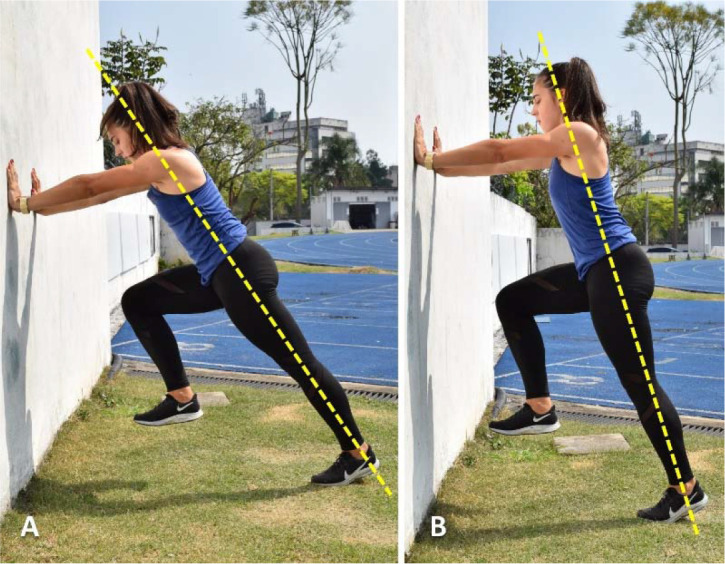
*. “Wall drive” executed with two different trunk inclinations (i.e., ≥ 45°), to simulate the sprinting posture adopted at different stages of the acceleration phase of sprinting: early-to-mid acceleration phase (Panel A); mid-to-late acceleration phase (Panel B). Sprint and jump coaches usually prescribe these drills over very short duration (e.g., 5–6 s), gradually increasing the movement speed as technical proficiency improves.

**Figure 4:**
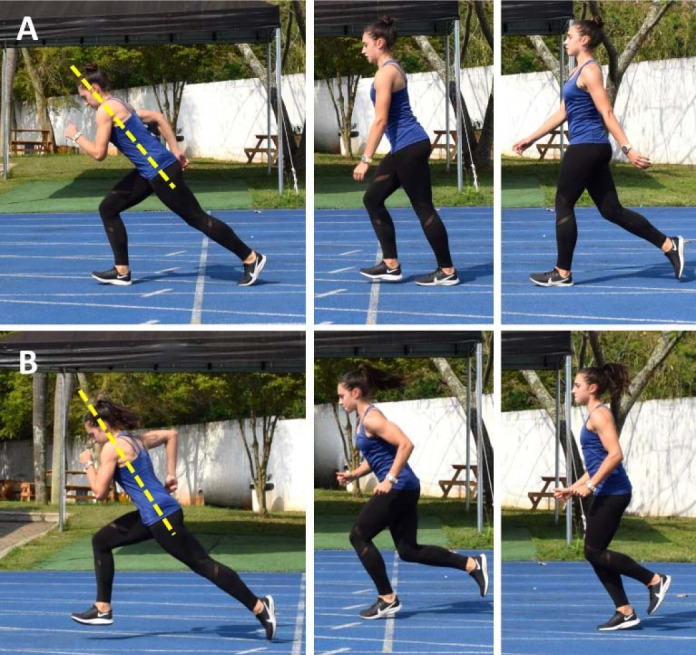
*. Acceleration drills performed from different starting speeds: the athlete initiates the drill by accelerating from a low speed (i.e., walking speed; Panel A); or from a moderate running speed (Panel B). Subsequently, the athlete must accelerate as much as possible over a short distance (e.g., ≤ 10 m). Coaches from various sports can incorporate these drills into their training programs, customizing the initial speeds to align with the specific acceleration-speed profiles of their respective sports. It is worth noting that lower starting speeds would result in greater degrees of trunk inclination at the beginning of the acceleration phase, while higher starting speeds will lead to a lower degree of trunk lean. *Note: *Sprint and jump coaches consistently prescribe technical drills as part of their daily warm-up routines, during both preparatory and competitive periods*.

The last secondary construct listed by expert sprint coaches for the drive/pick-up phase—also highlighted as a high-order construct along with the hip position for the maintenance phase—is posture (Jones et al., 2009). Indeed, posture is considered an important factor in every sprint phase (i.e., start, drive/pick-up, and maintenance phase), not only by track and field coaches, but also by researchers and sport scientists (Jones et al., 2009; Ma, 2023; Waters et al., 2019). Consequently, there is a substantial body of research on sprinting posture and the body position, with most studies subdividing this technical element into multiple subfactors (Donaldson et al., 2020; Kugler and Janshen, 2010; Ma, 2023; Miyashiro et al., 2019; Simperingham et al., 2016). However, in most sports, sprinting posture should be trained with a focus on the movements and actions that occur during the two principal running phases: acceleration and near to maximal/maximal speed phases. As mentioned earlier, these phases regularly occur in various sports, making the proper development of acceleration and top-speed postures (and qualities) essential for athletes of different disciplines (Nicholson et al., 2022; Oliva- Lozano et al., 2023; Zabaloy et al., 2023). In a more general perspective, sprint acceleration may be divided into early and late acceleration phases. During early acceleration, athletes usually adopt a more horizontally-oriented trunk position (i.e., mean trunk-lean measured from horizontal at the take-off, from ~25° to 45°) (Atwater, 1982; Hong and Bartlett, 2008), which naturally increases the ground contact time and facilitates the application of force in the horizontal direction (Atwater, 1982; Hong and Bartlett, 2008; Moir et al., 2018; Wild et al., 2011). The horizontal trunk lean is gradually reduced throughout this phase as speed increases, until achieving a mean value > 80° at the top-speed phase (Atwater, 1982; Hong and Bartlett, 2008). Therefore, to optimize sprinting posture at a range of speeds, coaches should prescribe technical drills that simulate the trunk position adopted during the distinct phases of sprint running, adjusting these drills according to the characteristics of each sport. For example, in soccer, where players usually initiate their sprints at from low-to-moderate speeds (Caldbeck and Dos’Santos, 2022b; Loturco et al., 2020b), coaches may prioritize acceleration drills performed in a more upright position (i.e., with a horizontal trunk lean > 45°) (Figures 3 and 4, Panel B). In addition to this, it is worth mentioning that elite sprinters exhibited a more vertical trunk orientation when compared to their sub-elite peers during the early acceleration phase (Donaldson et al., 2020), thereby offering valuable insights for selecting more efficient training drills to enhance acceleration capabilities in team-sport athletes.

For expert sprint coaches, the hip position refers to the athletes’ ability to maintain a high center of mass with a slight anterior pelvic tilt while sprinting (Donaldson et al., 2020; Thompson et al., 2009). In fact, hip and trunk positions are closely interconnected and it has been suggested that excessive forward trunk lean could impact the athlete’s capacity to achieve the necessary hip joint motion during the acceleration phase (Jones et al., 2009; Thompson et al., 2009). An adequate trunk lean is essential to adopt an optimal body posture, allowing the correct positioning of the lower limb segments and, thus, effective force application throughout this phase (Donaldson et al., 2020; Hong and Bartlett, 2008). Furthermore, the trunk, including the pelvis, plays a crucial role in controlling the amount of body rotation around the transverse axis when the athlete is in contact with the ground (Hong and Bartlett, 2008). This role becomes even more critical during the transition from acceleration to near to maximal/maximal speed phases, a period in which the magnitude and orientation of vertical and horizontal force components change drastically (Hong and Bartlett, 2008; Wild et al., 2011). Over the acceleration period, the center of mass rotates forward around the stance foot before the rapid extension of the stance leg (Jacobs and Van Ingen Schenau, 1992; Wild et al., 2011). Without an efficient rotation, the center of mass would assume a more vertical trajectory, compromising the orientation of ground reaction forces in the horizontal direction and thus affecting acceleration efficiency (Wild et al., 2011). Hence, proper pelvic rotation combined with correct trunk positioning is fundamental to place the athlete in a more favorable position to rapidly extend their legs and apply force horizontally (Wild et al., 2011). In light of this, and despite being perceived as different constructs for different sprint running phases, the hip position, the trunk position, leg extension, and the sprinting posture are interconnected technical elements that may (and should) be trained simultaneously. Figures 1–4 present some drills that can be used for this purpose, each accompanied by a detailed description and a respective explanatory note.

### Resistance Training and Complex Training

Overall, around 45% of Brazilian Olympic sprint and jump coaches identified resistance training as a primary method for speed development. These practitioners usually combine a variety of strength-power exercises (e.g., squat, jump squat, Olympic weightlifting, hip-thrust, etc.) in the same training session, across a comprehensive range of loads (i.e., 40–90% 1RM). The volume, frequency, and intensity of these sessions vary considerably from the preparatory to the competitive period, with certain adjustments and shift of emphasis according to the specific characteristics and immediate needs of athletes (e.g., focusing on the development of acceleration or top-speed qualities). In addition, 21% of these coaches prescribe resistance exercises as part of complex training methods, combined with a variety of speed-power drills. Considering the complexity and relevance of these topics, we decided to include a third article on resistance training practices in this track and field collection. This article will be published sequentially in the *Journal of Human Kinetics*.

## Conclusions

Olympic sprint and jump coaches have great expertise in training and producing faster athletes. To achieve this goal, they utilize a wide range of speed training methods, both in isolated and combined forms. Six of these methods are listed and described here. Due to its specificity, MSS training is the method most commonly used by these experts to enhance sprinting speed. Additionally, it could be seen as a good strategy for mitigating the risk of muscle injuries, particularly in the hamstrings, which is always a significant concern for team-sport players. Resisted sprinting is primarily employed to improve acceleration ability, under light and moderate loading conditions (i.e., 10% to 30% V_dec_ or 20% to 50% BM), with the main intention of resembling proper sprinting technique. Overspeed running is implemented as a complementary method to enhance top-speed qualities during the in-season period or during phases of reduced training volume (i.e., tapering), with assistance loads not exceeding 10% of the maximal sprint speed achieved by athletes within the target sprint zone. Uphill and downhill running are utilized to artificially introduce additional resistance or increased sprint speed into speed-specific training programs. Extreme slopes (i.e., >10% for uphill and >7% for downhill sprints) should be avoided to minimize abrupt changes in the sprinting posture and technique, as well as acute decrements in sprint speed. In general, inclinations between 5 and 10% resemble the technical adjustments related to the early acceleration phase, whereas smoother gradients (< 5%) reflect the movements observed in the late acceleration phase. Sport-specific movements are often trained using weighted vests, in an attempt to simulate sprint-specific actions with extra resistance. However, according to the literature, this method appears to be ineffective for optimizing linear sprint performance, especially in highly-trained athletes. Instead, it should preferably be applied to increase COD speed in athletes from multidirectional sports (e.g., team-sports), with overloads ≤ 20% BM. Form running is prescribed in the form of technical drills throughout the entire training season, predominantly as part of the traditional warm-up routine of these track and field athletes. These drills seem to be effective in enhancing the sprinting posture and technique and may be used within different contexts, for different purposes. Coaches and practitioners can utilize the methods and strategies detailed here to create efficient training programs for their athletes in an era when sprint speed is becoming increasingly important across multiple sports.
